# Clinical features and “early” corticosteroid treatment outcome of pediatric *mycoplasma pneumoniae* pneumonia

**DOI:** 10.3389/fcimb.2023.1135228

**Published:** 2023-04-04

**Authors:** Jinrong Liu, Ruxuan He, Xiaoyan Zhang, Fei Zhao, Liyong Liu, Heng Wang, Shunying Zhao

**Affiliations:** ^1^ Department of Respiratory Medicine, China National Clinical Research Center of Respiratory Disease, Beijing Children’s Hospital, National Center for Children’s Health, Capital Medical University, Beijing, China; ^2^ National Institute for Communicable Disease Control and Prevention, Chinese Center for Disease Control and Prevention, State Key Laboratory of Infectious Disease Prevention and Control, Beijing, China

**Keywords:** *Mycoplasma pneumoniae*, pneumonia, corticosteroid, outcome, children

## Abstract

**Background:**

Many children with *mycoplasma pneumoniae* (MP) pneumonia (MPP) developed sequelae such as bronchiolitis/bronchitis obliterans (BO). Early corticosteroid therapy might prevent disease progression. This study aimed to use “early” corticosteroid and observe the treatment outcome in patients with MPP.

**Methods:**

Patients who had pulmonary infiltrations on chest imaging within 5 days of the disease course and were suspected of having MP infection on admission were enrolled. Among them, patients whose disease course was within 10 days on admission were ultimately enrolled. We analyzed their data including the clinical features, the starting time and dose of corticosteroid therapy, and the treatment outcome. According to chest imaging, we divided patients into two groups (Group A: bronchiolitis-associated lesions or ground-glass opacities; Group B: pulmonary segmental/lobar consolidation).

**Results:**

A total of 210 patients with confirmed MPP were ultimately enrolled. There were 59 patients in Group A and 151 patients in Group B. Patients in Group A were more prone to have allergy histories, hypoxemia, wheezing sound, and wet rales on auscultation than those in Group B. Corticosteroid treatment was initiated between 5 and 10 days of disease onset in all patients and 6–7 days in most patients. Methylprednisolone was prescribed in all patients within 10 days of disease onset, and the highest prescribed dose was at least 2 mg/kg/day. In Group A, methylprednisolone >2 mg/kg/day was prescribed in 22 patients, and among them, 8 patients with diffuse bronchiolitis-associated lesions received high-dose methylprednisolone therapy. After 3 months, lung CT revealed slightly segmental ground-glass opacity in three patients. In Group B, methylprednisolone >2 mg/kg/day was prescribed in 76 patients, and among them, 20 patients with pulmonary lobar consolidation received high-dose methylprednisolone therapy. After 3 months, chest imaging revealed incomplete absorption of pulmonary lesions in seven patients. Among them, five patients with consolidation in more than one pulmonary lobe ultimately had slight BO.

**Conclusion:**

In hospitalized patients with MPP, particularly severe MPP, the ideal starting time of corticosteroid treatment might be 5–10 days, preferably 6–7 days, after disease onset. The initial dosage of corticosteroid therapy should be decided according to the severity of the disease. MPP patients with diffuse bronchiolitis-associated lesions/whole lobar consolidation on imaging might require high-dose corticosteroid therapy.

## Introduction

*Mycoplasma pneumoniae* (MP) is a major pathogen of pediatric community-acquired pneumonia. Many refractory, severe, fulminant, or even fatal cases who were not responsive to macrolide antibiotics or susceptible antibiotics have been reported mainly in east Asia (Lee et al., 2006; Tamura et al., 2008; You et al., 2014; Liu et al., 2018; Liu et al., 2018; Yang et al., 2019; Zhang et al., 2021). The host’s cell-mediated immunity plays a key role in the development of pulmonary lesions in MP pneumonia (MPP). It is confirmed that corticosteroid could effectively initiate the rapid improvement of clinical symptoms and chest radiographic findings (Lee et al., 2006; Tamura et al., 2008; You et al., 2014; Yang et al., 2019). However, corticosteroid resistance has been reported in some MPP patients with more serious radiological findings (Yan et al., 2016; Liu et al., 2019). Successful methylprednisolone pulse therapy (30 mg/kg/day) has been reported in some patients with severe MPP (SMPP) (Tamura et al., 2008; Shen et al., 2013; You et al., 2014; Liu et al., 2019). Unfortunately, many patients with refractory MPP (RMPP) and SMPP inevitably developed sequelae, mainly atelectasis, bronchiectasis, and bronchiolitis obliterans due to bronchiolitis-associated lesions (Zhao et al., 2017; Wen et al., 2018) and bronchitis obliterans due to pulmonary consolidation (Leong et al., 1997; Zhao et al., 2017; Liu et al., 2018; Liu et al., 2019), although they were treated with susceptible antibiotics, high-dose corticosteroid, and bronchoscopy lavage therapy (BLT).

Yang et al. reported that early corticosteroid therapy might prevent disease progression, and anti-MP antibiotics might have limited effects on MPP (Yang et al., 2019), which was consistent with our clinical experience. However, the definition of “early” is unclear. In addition, there is a wide range of methylprednisolone (1–30 mg/kg/day) doses in the treatment of MPP (Tamura et al., 2008; Yang et al., 2019). Therefore, many factors of MPP about corticosteroid therapy remain unclear, such as which day the optimal starting time is, and how much the ideal initial dose is. Elucidating these issues is important to help guide standard treatment and reduce sequelae.

During the autumn and winter epidemic in 2019 in North China, to improve the prognosis and avoid the sequelae (Zhao et al., 2017; Liu et al., 2018; Liu et al., 2019), we had the plan to use “early” corticosteroid empirically for MPP patients who had pulmonary infiltrations on imaging within 5 days of disease course, and the dose of methylprednisolone was decided according to the severity of the disease. In addition, the clinical features of MPP were reviewed.

## Methods

### Study population and definitions

Patients who were admitted to the Department of Respiratory Medicine at Beijing Children’s Hospital, between July 2019 and January 2020, and who had pulmonary infiltrations on chest imaging within 5 days of disease course and were suspected of having MP infection on admission were enrolled. Among them, patients whose disease course was within 10 days on admission were ultimately enrolled. In this study, disease course was calculated as the duration of fever.

**Figure 1:**
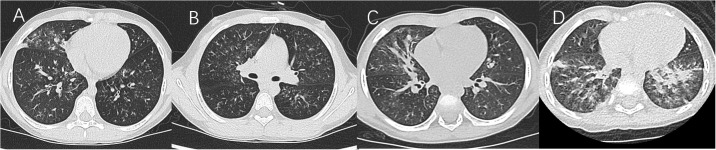
**(A, B)** Case 1. **(C)** Lung HRCT showed unilateral/bilateral inflammatory bronchiolitis including typical diffuse tree-in-bud signs and centrilobular nodules. **(D)** Lung HRCT showed diffuse high-density ground-glass opacification in bilateral lungs.

In the nearly 5 years, younger children with MPP and more children with MP-associated bronchiolitis were found in North China. The main sequelae of MPP are bronchiolitis obliterans (Zhao et al., 2017; Wen et al., 2018) and bronchitis obliterans (Liu et al., 2018; Zhao et al., 2020), which are mainly caused by bronchiolitis-associated lesions and pulmonary consolidation, respectively, according to Guidelines for diagnosis and treatment of pediatric MPP in China (Version 2023, published by National Health Commission of the People’s Republic of China, http://www.gov.cn/zhengce/zhengceku/2023-02/16/content_5741770.htm). Therefore, patients were classified into two groups in this study. Group A was defined as having chest high-resolution CT (HRCT) results that mainly revealed centrilobular nodules, branching linear structures, tree-in-bud signs, bronchiolar wall thickening, and ground-glass opacities (**Figures 1A, B** in a patient numbered Case 1, extremely typical diffuse bronchiolitis-associated lesions on imaging; **Figures 1C, D**). Group B was defined as having chest x-ray/HRCT results that mainly revealed pulmonary segmental/lobar consolidation.

### Diagnostic criteria

In this study, MP infection met both of the following criteria: (1) serum anti-MP IgM titer ≥1:320, or/and the titer of anti-MP IgM increased by four times or more in the recovery and acute stage; (2) positive results on MP polymerase chain reaction (PCR) testing of pharyngeal swab. SMPP was defined as MPP with one of the following (Subspecialty Group of Respiratory Diseases, The Society of Pediatrics et al., 2013): (1) increased respiratory rate, (2) dyspnea and cyanosis, (3) multilobe involvement or ≥2/3 lung involvement, (4) extrapulmonary complication, (5) pleural effusion, and (6) pulse oxygen saturation in room air ⩽92%.

### Etiological detection and data collection

After admission, all patients were subjected to the following: MP antibody identification (at least twice), MP-PCR testing of pharyngeal swab, and nasopharyngeal aspirate/swab for common respiratory tract virus antigens (respiratory syncytial virus, adenovirus, and influenza virus). In addition, MP genotyping and antimicrobial susceptibility testing, and next-generation sequencing (NGS, including metagenomic and metatranscriptomic analyses) in bronchoalveolar lavage fluid (BALF)/sputum/pleural effusion were performed in some patients.

The demographic and clinical data were collected and recorded for each patient. The findings of chest imaging and bronchoscopy, the starting time and dose of corticosteroid therapy, and treatment outcome were recorded.

### MP genotyping and antimicrobial susceptibility testing, and NGS testing

Each BALF sample (1.5–2 ml) was stored and frozen at −20°C for 1 month, which would be subject to MP genotyping and antimicrobial susceptibility testing. Occasionally, another redundant 1.5 ml of BALF sample in a patient (we numbered her Case 2) of Group B was transported in a hot summer day at 35°C for 5 min and subsequently stored at 4–8°C for 50 h and then frozen at −20°C for 1 month. The above testing was performed as previously reported (Zhao et al., 2019). In the meantime, BALF/pleural effusion/sputum samples were stored at 4–8°C and transported to the NGS laboratory within 36–50 h in some patients whose parents agreed with NGS detection.

### Numbered cases

Children with sequelae of bronchiolitis/bronchitis obliterans (BO) were numbered from Case 3, except the above Cases 1 and 2.

### Statistical analyses

SPSS version 17.0 was used for statistical analyses. All statistical hypothesis tests were two-sided, and *p*-values < 0.05 were considered statistically significant.

## Results

### Study population

**Figure 2:**
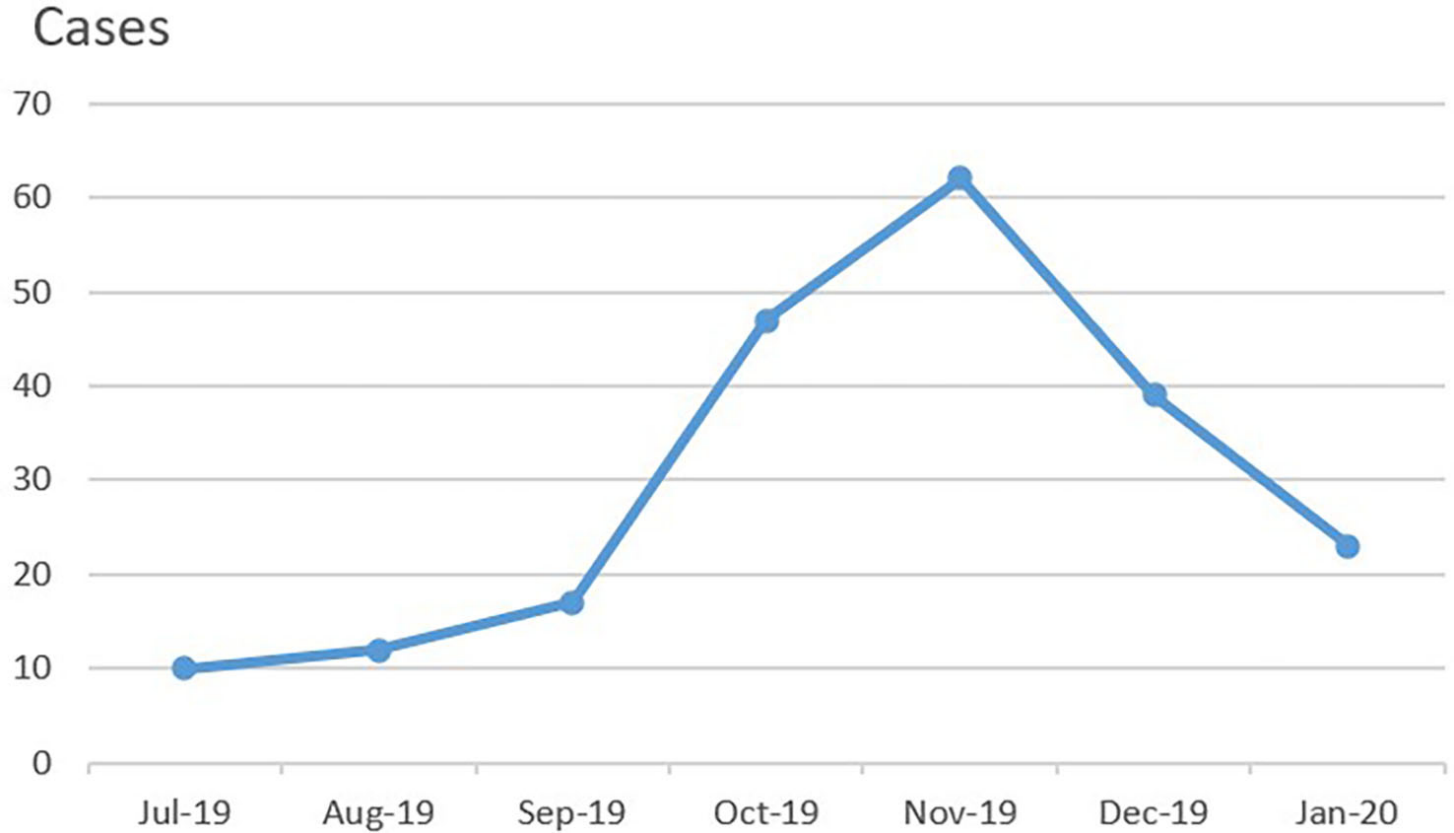
The monthly enrolled case number of MPP between July 2019 and January 2020.

A total of 217 patients who had pulmonary infiltrations on imaging within 5 days of disease course were initially suspected of having MPP on admission between July 2019 and January 2020. Among them, 210 patients with confirmed MPP (age range: 1 year 3 months to 16 years 1 month) were ultimately enrolled in this study (**Table 1**). The duration of disease before hospitalization was 5–10 days in all patients, 6–7 days in most, and 5 days in one patient (Case 3, Group B, **Tables 2**, **3**). Most patients resided in Beijing. 54.3% (*n* = 114) were male, and 45.7% (*n* = 96) were female. As shown in **Figure 2**, the number of patients enrolled in November 2019 was the most followed by October 2019. A total of 36 patients had confirmed or suspected MP infection contact history with their family members or classmates. History revealed allergic diseases in 35 patients, febrile seizures in 3 patients, Kawasaki disease in 2 patients, idiopathic thrombocytopenic purpura in 2 patients (including Case 4, Group B), ventricular septal defect in 1 patient, infectious mononucleosis in 1 patient, and brain germ cell tumor in 1 patient who had had received chemotherapy (Case 1, Group A).

**Table 1:** Clinical characteristics and laboratory data on admission in MPP patients.

Project	Group A (*n* = 59)	Group B (*n* = 151)	
Age ≤3 years	25.4% (15/59)	9.3% (14/151)	*p* < 0.05
Male:female ratio	34:25	80:71	*p* > 0.05
Allergy history	30.5% (18/59)	11.3% (17/151)	*p* < 0.05
Fever duration before admission (days)	6.91 ± 3.37	6.32 ± 2.88	*p* > 0.05
Peak temperature (°C)	38.4–41	38.3–41	–
Hypoxemia	20.3% (12/59)	15.9% (24/151)	*p* > 0.05
Rash	0% (0/59)	2.0% (3/151)	*p* > 0.05
Wheezing sound	25.4% (15/59)	7.3% (11/151)	*p* < 0.05
Wet rales	69.5% (41/59)	35% (36/151)	*p* < 0.05
Respiratory failure	13.6% (8/59)	7.3% (11/151)	*p* > 0.05
Encephalitis	0.0% (0/59)	1.3% (2/151)	*p* > 0.05
WBC (×10^9^/L)	7.59 ± 2.26 (4.08–12)	8.14 ± 3.47 (2.98–18.31)	*p* > 0.05
CRP (mg/L)	18.02 ± 15.75 (6–78)	28.50 ± 32.52 (5–190)	*p* < 0.05
CRP > 8 mg/L within 5 days	40.7% (24/59)	55.0% (83/151)	*p* > 0.05
CRP > 30 mg/L within 5 days	8.5% (5/59)	12.6% (19/151)	*p* > 0.05
LDH (IU/L)	308.57 ± 101.45 (214–785)	436.72 ± 606.05 (227–6,025)	*p* < 0.05
D-dimer (µg/L)	1,011.57 ± 1,367.34 (231–7,983)	1,371.11 ± 1,971.53 (200–10,000)	*p* > 0.05
Lung function (*n*)	15	22	
Normal (*n*)	2	3	*p* > 0.05
OVD (*n*)	13	19	*p* > 0.05
SMPP	72.9% (43/59)	53.6% (81/151)	*p* < 0.05
ICU for hospitalization	0.0% (0/59)	2.0% (3/151)	*p* > 0.05

WBC, white blood cells; CRP, C-reactive protein; LDH, lactate dehydrogenase; OVD, obstructive ventilation dysfunction; SMPP, severe *mycoplasma pneumoniae* pneumonia; ICU, intensive care unit.

**Table 2:** Treatment and clinical outcomes in MPP patients.

Project	Group A (*n* = 59)	Group B (*n* = 151)
Starting time of corticosteroid		
5 days of disease course	0	2 (Cases 3 and 6)
6 days of disease course	26 (including Case 1)	71
7 days of disease course	29(including Case 2)	58 (including Case 5)
8 days of disease course	3	16 (including Case 4)
9 days of disease course	1	3
10 days of disease course	0	1 (Case 7)
Highest dose of methylprednisolone		
2 mg/kg/day	37	75 (including Case 2)
>2, <5 mg/kg/day	14	56
5 mg/kg/day	4	8
10 mg/kg/day	3 (including Case 1)	8
15 mg/kg/day	1	–
20 mg/kg/day	–	3
30 mg/kg/day	–	1
IVIG	6	9
BLT	41	120
CPAP	9	8
Mechanical ventilation	0	1 (Case 5)
Clinical outcomes		
Bronchiolitis obliterans	0	2
Bronchitis obliterans	0	3

IVIG, intravenous immunoglobulin; BLT, bronchoalveolar lavage therapy; CPAP, continuous positive airway pressure.

**Table 3:** The dose and timing of methylprednisolone treatment in the patients with sequela (Cases 3–7).

Days of illness onset	Peak temperature (°C)/CRP (mg/L)/Methylprednisolone (mg/kg/day)				
	Case 3 (8 years 8 months, girl)	Case 4 (7 years, girl)	Case 5 (5 years 8 months, girl)	Case 6 (3 years 9 months, boy)	Case 7 (7 years, girl)
2	38.3/12/0	40.0/20/0	38.4/11/0	38.9/NA/0	39.9/NA/0
3	40.1/9/0	39.9/61/0	38.7/NA/0	39.6/10/0	39.8/NA/0
4	41.6/169/0	40.1/169/0	39.5/35/0	39.9/NA/0	39.7/41/0
5	42/179/5	40.0/216/0	40.3/71/0	40.2/55/1	40.5/NA/0
6	39.9/189/20	39.8/239/0	40.1/179/0	39.9/NA/2	39.8/67/0
7	38.8/NA/30	40.0/196/0	40.2/256/2	40/NA/2.5	39.2/NA/0
8	38.5/NA/30	40.0/NA/4	39.2/230/2	40.1/102/20	38.7/NA/0
9	38.5/49/10	39.5/198/12	39.1/NA/2	38.1/NA/20	38.2/36/0
10	37.9/22/5	39.5/NA/20	39.0/238/10	38.8/NA/10	36.6/NA/2
11	38.4/NA/2	38.5/138/20	38.8/22010	37.3/76/5	36.5/20/2
12	37.0/NA/2	38.0/NA/20	38.7/160/10	37.7/55/2	36.3/NA/2
13	36.6/3/2	37.5/72/10	39.1/91/20	37.3/12/2	36.5/12/2

Cases 3–7: before admission to our department in yellow; after admission to our department in blue. NA, not available.

### Clinical characteristics and laboratory data

All patients presented with fever and deep wet cough (non-productive cough). Sputum was not easily coughed up. Most coughed with white sticky sputum. Only two coughed with yellow sticky sputum, and NGS only detected MP in BALF on them. 20.3% of Group A and 15.9% of Group B had hypoxemia (**Table 1**). Two patients accompanied with encephalitis (Group B). Other clinical symptoms and inflammatory markers (on admission) are shown in **Table 1**. It is worth noting that patients with wet rales or wheezing sounds on auscultation were mostly observed in Group A and they often had a history of allergies (**Table 1**).

### Chest imaging findings

In Group A, HRCT revealed unilateral or bilateral bronchiolitis-associated lesions in all patients. Chest x-ray revealed patchy shadow in most patients. One patient accompanied with mild pleural effusion.

**Figure 3:**
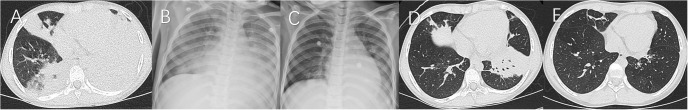
Case 3. Chest imaging showed bilateral/unilateral high-density lesions (**A**, day 6; **B**, day 11; **C**, day 16; **D**, day 21), and string-atelectasis and slight enlargement of bronchial lumen (**E**, 11 months after illness onset).

**Figure 4:**
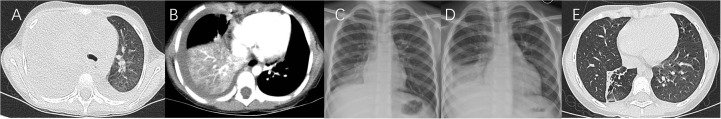
Case 4. Chest imaging showed right high-density lesions and left pleural effusion (**A**, day 8), right necrotizing pneumonia and pleural effusion (**B**, day 17), right high-density lesions (**C**, day 23; D, day 33), right pleural effusion **(D)**, and right localized atelectasis and bronchiectasia (**E**, 20 months after illness onset).

**Figure 5:**
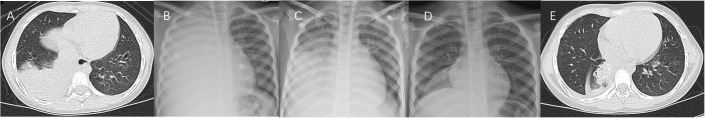
Case 5. Chest imaging showed right high-density lesions (**A**, day 4; **B**, day 8; **C**, day 10) and right pleural effusion **(B, C)**, and right localized atelectasis (**D**, day 70; **E**, 19 months after illness onset).

**Figure 6:**
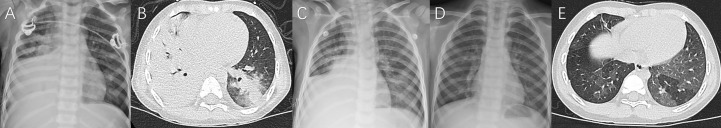
Case 6. Chest imaging showed bilateral/unilateral high-density lesions (A, day 9; B, day 10; C, day 11), right pleural effusion **(A–C)**, no abnormality (**D**, day 24), and the signs of bronchiolitis obliterans-associated mosaic perfusion (**E**, 9 months after illness onset).

In Group B, chest imaging revealed segmental/lobar consolidation in all the 151 patients, obstructive emphysema in 2 patients (including Case 2), and a small amount to massive pleural effusion in 19 patients (including Cases 3–6, **Figures 3**–**6**). Contrast-enhanced lung CT was performed in four patients and revealed pulmonary artery thrombosis in two patients (**Table 4**).

**Table 4:** The results of chest imaging, bronchoscopy, and pathogen test in MPP patients.

Project	Group A (*n* = 59)	Group B (*n* = 151)	
Pleural effusion	1	19	*p* < 0.05
Pulmonary artery thrombosis	0	2	*p* > 0.05
Bronchoscopy findings	41	120	*p* > 0.05
Mucus plugs	3	23	*p* < 0.05
Bronchial cast	1	9	*p* > 0.05
Mucosal follicles	16	49	*p* > 0.05
Mucosal necrosis	1	21	*p* < 0.05
Negative rate of MP-IgM on admission	64.4% (38/59)	66.2% (100/151)	*p* > 0.05
MP-genotyping			
Genotype 1	73.2% (30/41)	71.7% (86/120)	*p* > 0.05
Genotype 2	22.0% (9/41)	24.2% (29/120)	*p* > 0.05
Genotypes 1 and 2	4.9% (2/41)	4.2% (5/120)	*p* > 0.05
MP-macrolide resistance	100%	100%	*p* > 0.05
Virus co-infection	5	5	*p* > 0.05
ADV	1	4	–
IV	1	1	–
RSV	1	0	–
HBoV	1	0	–
HHV-6A	1 (Case 1)	0	–
Bacteria co-infection	1 (*Sp*)	3 (*Hi*, *Pa*, *Mc*)	*p* > 0.05

ADV, adenovirus; IV, influenza virus; RSV, respiratory syncytial virus; HBoV, human bocavirus; HHV, human herpesvirus; MP, *mycoplasma pneumoniae*; *Sp, Streptococcus pneumoniae; Hi, Haemophilus influenzae, Pa, Pseudomonas aeruginosa, Mc, Moraxella catarrhalis*.

### Bronchoscopy therapy and findings

In fact, in group A, bronchoscopy was carried in 41 petients and bronchoscopy revealed swollen bronchial mucosa and extensive increased sticky secretions at the early stage in these 41 patients (**Tables 2**, **4**). Among them, bronchoscopy revealed mucosal follicles in 16 patients, mucus plugs in 3 patients, bronchial cast in 1 patient, and mucosal necrosis in 1 patient (**Table 4**). Bronchoscopy was carried out two times in five patients.

In fact, in Group B, bronchoscopy was carried out in 120 patients and revealed swollen bronchial mucosa and increased sticky secretions at the early stage in these 120 patients (**Tables 2**, **4**). Among them, bronchoscopy revealed mucosal follicles in 49 patients, mucus plugs in 23 patients, mucosal necrosis in 21 patients, and bronchial cast in 9 patients (**Table 4**). Bronchoscopy was carried out at least two times in 58 patients. At the late stage, bronchoscopy revealed stenosis in 12 patients and obliteration of bronchial segments in 5 patients (Cases 3–7).

It is worth noting that few patients with SMPP, particularly in Group A, had transient faster breathing and more severe hypoxia within 12 h after BLT.

### Pathogen test results

#### Negative serum MP-IgM rate on admission

The negative rate of MP-IgM on admission was 64.4% and 66.2% in Group A and Group B, respectively (**Table 4**).

#### MP genotyping, antimicrobial susceptibility testing, and MP culture in BALF/pleural effusion

In Group A, genotype 1, genotype 2, and combined genotype 1 and genotype 2 were detected in the BALF of 30 (73.2%), 9 (22.0%), and 2 (4.9%) patients, respectively.

In Group B, genotype 1, genotype 2, and combined genotype 1 and genotype 2 were detected in the BALF of 86 (71.7%), 29 (24.2%), and 5 (4.2%) patients, respectively. Genotype 1 was detected in the pleural effusion of 2 patients (Cases 3 and 4).

The MP culture-positive rate of BALF specimens was 100%. All strains were macrolide-resistant (MR) [including 2 BALF samples (different storage temperature) with a similar PCR CT value in Case 2], and carried the A2063G mutation in 160 patients and A2064G mutation in 1 patient (Group B). No strains with resistance to the levofloxacin and tetracycline were identified.

#### Virus/bacterial co-infection and NGS testing

Virus and bacterial co-infection are shown in **Table 4**, and suggested the low co-infection in the early stage of MPP. The NGS (metagenomic and metatranscriptomic analyses) performed revealed positive MP-DNA and MP-RNA in BALF of 13 patients, pleural effusion of 2 patients, and sputum sample of 1 patient.

### Treatment and clinical outcomes

Continuous positive airway pressure was administered in nine patients of Group A and eight patients of Group B (**Table 4**). One patient (Case 5, Group B) received treatment with mechanical ventilation. Starting time of macrolides was within 5 days of disease onset in 57 patients of Group A and 113 patients of Group B. Sensitive anti-MP antibiotics (minocycline or moxifloxacin) was ultimately administered to 5 patients of Group A and 19 patients of Group B. Methylprednisolone was prescribed in all patients on admission (5–10 days after disease onset) and the highest dose was at least 2 mg/kg/day (**Table 2**). Corticosteroid treatment was initiated at 6–7 days of disease course in most patients (*n* = 184, **Tables 2**, **3**). The highest prescribed dose of methylprednisolone was for 3 days or so and then was gradually withdrawn (decreased to half dose every 3 days or so) mainly based on the body temperature. Intravenous immunoglobulin was used in six patients of Group A and nine patients of Group B (**Table 2**). In addition, low-molecular-weight heparin was administered in patients whose D-dimer was higher than 3,000 µg/L. Only a few patients had secondary or recurrent lower respiratory infections after discharge.

In Group A, methylprednisolone >2 mg/kg/day was prescribed in 22 patients (**Table 2**). Among them, eight with bilateral diffuse bronchiolitis received high-dose methylprednisolone therapy (5 mg/kg/day, 10 mg/kg/day, and 15 mg/kg/day in four patients, three patients, and one patient, respectively) (**Table 2**). Pulmonary function was performed in the recovery stage in 15 patients and revealed mild to moderate obstructive ventilation dysfunction in 13 patients (**Table 1**). After 3 months, HRCT revealed no abnormalities in 56 patients and slightly segmental ground-glass opacity in 3 patients.

In Group B, methylprednisolone >2 mg/kg/day was prescribed in 76 patients (**Table 2**). Among them, 20 patients with lobar consolidation received high-dose methylprednisolone therapy (5 mg/kg/day, 10 mg/kg/day, 20 mg/kg/day, and 30 mg/kg/day in 8 patients, 8 patients, 3 patients, and 1 patient, respectively). Pulmonary function was performed in the recovery stage in 22 patients and revealed mild to moderate obstructive ventilation dysfunction in 19 patients (**Table 1**). After 3 months, chest imaging revealed incomplete absorption of pulmonary lesions in seven patients. Among them, five patients ultimately had slight bronchitis obliterans (Cases 3–5, **Figures 3**–**5**) and bronchiolitis obliterans (Case 6, **Figure 6**; Case7). The timing and dose of methylprednisolone treatment in Cases 3–7 are shown in **Table 3**. Cases 3–6 had respiratory failure, and moderate to massive pleural effusion. Cases 3 and 5 had abdominal pain before high-dose corticosteroid treatment. Fifty-seven days after illness, MP loads were still high, up to 3×10^8^ copies/ml, in the BALF of Case 5, and she received sirolimus therapy for 1 month. On 19 months after illness, MP-DNA (117 of unique reads) in NGS was still positive and MP-RNA in PCR was negative in BALF; serum MP-IgM was positive with a titer of ≥1:320 in Case 5. The body temperature dropped to normal after being treated with minocycline before admission in Case 7; however, pulmonary consolidation did not improve on her and she received corticosteroid therapy on admission (10 days of disease course). To date, Cases 3–7 were asymptomatic, which further suggested that sequelae had been minimized.

## Discussion

Pediatric MPP is a significant public health problem in China, and it is typically mild and even self-limited. However, in recent years, more and more patients with SMPP, RMPP, bronchiolitis-associated MPP, and MPP-associated sequela have been reported (Zhao et al., 2017; Wen et al., 2018; Liu et al., 2019; Liu et al., 2020; Zhang et al., 2021), posing great challenges to pediatricians. It is mandatory to recognize RMPP and SMPP in the early stage, and timely effective anti-MP therapy and immunomodulating therapy are the main strategies for RMPP (Tong et al., 2022). However, the negative rate of anti-MP-IgM at the early stage was high, up to 66.2% (**Table 4**), and the MP-PCR test takes time and is not be available in some hospitals; thus, early pathogenic diagnosis cannot be achieved in some patients. Therefore, early recognition of the clinical features of MPP, rapid MP-PCR testing, and timely empirical therapy are extremely important.

MP type 1 was the predominant genotype from 2008 to 2012 in Beijing, and a shift from type 1 to type 2 began to occur in 2013 (Zhao et al., 2015). In our present study, MP type 1 was still the main genotype; however, the rate of MP genotype 2 increased to 24.2% (**Table 4**) higher than our previous study (Liu et al., 2019; Guo et al., 2022). In addition, 7 patients had co-infection with type 1 and type 2 MP isolates (**Table 4**), which has been reported in only a few studies (Zhao et al., 2015; Li et al., 2021). The total MR MP rate was high, up to 100% (**Table 4**), which was consistent with the previously reported rate (Liu et al., 2019; Zhao et al., 2020; Guo et al., 2022). Positive MP culture in the BALF of Case 2 and positive MP-RNA in samples of patients who received metatranscriptomic sequencing testing suggested that MP could be easy to survive *in vitro*, because these samples had been stored at 4–8°C for at least 36 h. Furthermore, the metatranscriptomic sequencing performed revealed positive MP-RNA (unique reads: 26 and 59) in the pleural effusion of two patients, which suggested that there could be a few live MP in pleural effusion that have not been reported previously. Most of our patients were treated with macrolides within 5 days and were unresponsive. MP was difficult to clear even by sensitive anti-MP antibiotics (Liu et al., 2019; Zhao et al., 2020), which may explain the long-term positive MP-IgM such as Case 5. The pathogenesis of MPP is mainly attributed to cell-mediated immunity and cytokine responses against the pathogens (Shimizu et al., 2011). Host genetic background was also found to be important for the severity of MPP (Fonseca-Aten et al., 2005; Meyer Sauteur et al., 2018). Therefore, corticosteroid therapy has been confirmed to be very important in hospitalized MPP patients (Liu et al., 2012; Chen et al., 2014; Yang et al., 2019).

Children under 3 years old were more easily prone to have bronchiolitis-associated MPP (Group A) than consolidation-associated MPP (Group B) (**Table 1**). Patients in Group A were more easily prone to have allergy histories and present with hypoxemia, wheezing sound, and wet rales on auscultation than those in Group B (**Table 1**), which was consistent with our pervious study (Zhao et al., 2017; Wen et al., 2018). Pleural effusion was more likely to occur in Group B (**Table 4**). In addition, inflammatory markers such as CRP and LDH in Group B were higher than that in Group A (**Table 1**), which suggested that inflammation and corticosteroid dose in Group B could be higher than that in Group A.

In this study, we found that pulmonary infiltration could occur within 5 days of illness in MPP, and rapid, serious, and even fulminant clinical deterioration occurred after 5 days of illness onset in our present (**Figures 3**–**6**) and reported studies (Liu et al., 2012). Therefore, there is an urgent need for lung ultrasound to dynamically detect lung lesions and prevent frequent radiation. The ideal starting time of corticosteroid treatment is 5–10 days after disease onset in SMPP (Version 2023, published by National Health Commission of the People’s Republic of China, http://www.gov.cn/zhengce/zhengceku/2023-02/16/content_5741770.htm). RMPP patients with fever >7 days, CRP >40 mg/L, and consolidation >2/3 pulmonary lobe have been suggested to receive a timely corticosteroid treatment (Liu et al., 2012). Fever ≥10 days, CRP ≥137 mg/L, and consolidation >2/3 pulmonary lobe may predict the occurrence of sequela in MPP (Predictive factors for sequela of bronchitis obliterans in refractory *mycoplasma pneumoniae* pneumonia, to be published in *Zhonghua Er Ke Za Zhi*). Methylprednisolone pulse therapy had been prescribed in 21 patients with SMPP; however, sequela of BO had not been avoided in 13 who received treatment with corticosteroid after 9 days of onset (Liu et al., 2019), which was consistent with our present Case 7 and suggested that the rapid irreversible airway remodeling could occur within 9 days of onset. Therefore, earlier corticosteroid treatment (<9 days even 7 days) should be initiated in SMPP (**Table 2**). Treatment with 2 mg/kg/day methylprednisolone may be ineffective in some patients with high fever >7 days, CRP ≥110 mg/L, and consolidation > whole pulmonary lobe (Chen et al., 2014). In our present study (**Table 3**), initial corticosteroid therapy on the 5 days of the disease course in Cases 3 and 6, and 1–5 mg/kg/day of initial methylprednisolone dose in Cases 3–7, failed to prevent the deterioration of the disease (higher CRP and more severe pulmonary injury), which further suggested that very early treatment (on day 5) and an insufficient starting dose of corticosteroid could not prevent a subsequent rapidly progressive pulmonary inflammatory storm in patients with ≥1 lobar consolidation. Therefore, the ideal starting time of corticosteroid treatment might be 6–7 days (important window period) of disease course (**Table 2**). Initial adequate dose of corticosteroid should be decided according to the severity of disease. Pulmonary infiltration particularly lobar consolidation and CRP > 30 mg/L (**Tables 1**, **3**) within 5 days of illness, and fever duration >5 days, were important clues for recognizing RMPP and SMPP. Persistent high fever, hypoxemia, CRP > 100 mg/L, diffuse bronchiolitis-associated lesions, or whole lobar consolidation on imaging (**Tables 2**, **3**, **Figures 3**–**6**) suggested that possible critical MPP would require corticosteroid pulse therapy.

MP persistent infection may cause airway obstruction and remodeling *in vitro* and animal experiments (Fonseca-Aten et al., 2005; Salvatore et al., 2008; Prince et al., 2018). Obstructive pulmonary function (**Table 1**) and the irreversible BO (Zhao et al., 2017; Liu et al., 2018; Wen et al., 2018; Liu et al., 2019; Zhao et al., 2020) suggested an early rapid airway injury and remodeling in children with SMPP. Therefore, timely and initial adequate dosage of corticosteroid therapy is extremely important in the window period, which can reduce airway inflammation and airway hypersecretion, and prevent rapid airway remodeling. Although the rate of MR MP was 100% in this study, macrolides were still used in most patients, because of their anti-inflammation and anti-remodeling properties (Kang et al., 2016). Sirolimus may also reduce airway inflammation and remodeling (Wang et al., 2020) and it might be a potential therapeutic drug (Liu et al., 2021); therefore, we prescribed sirolimus for 1 month in the recovery stage of Case 5.

The bronchoscopy performed revealed extensive increased sticky secretions in 41 patients of Group A, which may explain the hypoxemia, wheezing, wet rales, and the subsequent possible sequela of bronchiolitis obliterans (Zhao et al., 2017; Wen et al., 2018). Mucus plugs and mucosal necrosis under bronchoscopy were more likely to occur in severe patients of Group B, suggesting subsequent possible atelectasis and bronchitis obliterans (Liu et al., 2018; Liu et al., 2019; Zhao et al., 2020). Therefore, timely and multiple BLT is also extremely important, which can remove airway secretions and prevent lumen obstruction.

In general, patients with MPP admitted in our hospital were more severe than those in most hospitals of China. In this present study, we analyzed the clinical features, “early” corticosteroid treatment outcome, and prognosis of 210 MPP children who had pulmonary infiltrations on imaging within 5 days after onset. Based on our rich clinical experience, the prescribed “early”/on admission (5–10 days, mainly 6–7 days of disease course) corticosteroid with different doses for patients with MPP successfully prevented deterioration in most patients and minimized the sequelae (**Table 2**). To the best of our knowledge, this is the first study to emphasize the importance of both starting time and the initial dose of corticosteroid treatment in MPP particularly in SMPP.

## Conclusions

Patients in Group A were more prone to have allergy histories, hypoxemia, wheezing sounds, and wet rales on auscultation than those in Group B. Pulmonary infiltration within 5 days of illness, fever duration >5 days, and elevated inflammatory markers such as CRP were important clues for recognizing RMPP and SMPP. In hospitalized children with MPP, particularly SMPP (except rare fulminant MPP), the ideal starting time of corticosteroid treatment might be 5–10 days, preferably 6–7 days (important window period) after disease onset. The initial adequate dosage of corticosteroid therapy was extremely important in SMPP and should be decided according to the severity of the disease. MPP patients with both persistent high fever and diffuse bronchiolitis-associated lesions/≥1 pulmonary lobar consolidation can be recommended to receive high-dose corticosteroid therapy (**Table 3**).

## Data availability statement

The raw data supporting the conclusions of this article will be made available by the authors, without undue reservation.

## Ethics statement

The studies involving human participants were reviewed and approved by The Ethics Committee of Beijing Children’s Hospital, Capital Medical University (No. 2017-23). Written informed consent to participate in this study was provided by the participants’ legal guardian/next of kin. Written informed consent was obtained from the individual(s), and minor(s)’ legal guardian/next of kin, for the publication of any potentially identifiable images or data included in this article.

## Author contributions

JL, XZ, and RH conducted the analysis and drafted and revised the initial manuscript. HW advised on the design of the analysis and revised the manuscript. FZ and LL performed MP-related testing, analyzed experimental data, and revised the manuscript. SZ conducted the analysis and revised the initial manuscript. All authors contributed to the article and approved the submitted version.
